# Supercritical Fluid Extraction of Bioactive Components from Apple Peels and Their Modulation of Complex I Activity in Isolated Mitochondria

**DOI:** 10.3390/antiox13030307

**Published:** 2024-03-01

**Authors:** Antonella Aresta, Nicoletta De Vietro, Pietro Cotugno, Ciro Leonardo Pierri, Lucia Trisolini, Carlo Zambonin

**Affiliations:** 1 Department of Biosciences, Biotechnology and Environment, University of Bari “Aldo Moro”, Via Orabona 4, 70125 Bari, Italy; nicoletta.devietro@uniba.it (N.D.V.); l.trisolini@ibiom.cnr.it (L.T.); carlo.zambonin@uniba.it (C.Z.); 2 Department of Chemistry, University of Bari “Aldo Moro”, Via Orabona 4, 70125 Bari, Italy; pietro.cotugno@uniba.it; 3 Department of Pharmacy, Pharmaceutical Sciences, University of Bari “Aldo Moro”, Via Orabona 4, 70125 Bari, Italy; ciro.pierri@uniba.it

**Keywords:** SFE, HPLC, apple peels, bioactive compounds, in vitro activity

## Abstract

Supercritical fluid extraction (SFE) was used to extract bioactive compounds from apple (*Malus domestica*) peel waste from three different Italian cultivars. The bioactive fractions were extracted applying a temperature of 60 °C and a pressure of 250 bar for 15 min with 20% ethanol as co-solvent, at a flow rate of 2 mL/min. The total polyphenol (TP), anthocyanin (TA), ascorbic acid (AA), and antioxidant activity contents (TACs) were measured, while chromatographic analyses were performed to highlight the differences between the extracts. The Stark cultivar had the highest levels of polyphenols, anthocyanins, and ascorbic acid, while the Royal Gala cultivar showed the highest total antioxidant activity. SFE extracts were then tested for their effect on the mitochondrial NADH–ubiquinone oxidoreductase (Complex I) activity on mitochondria isolated from human embryonic kidney cells (HEK239). The Stark extract showed the most positive response in terms of NADH oxidation. The results obtained in this work highlight the potential of apple peel waste as a source of functional phytocompounds and suggest that Stark cultivar extracts may be exploited for pharmacological applications. This study supports the circular bioeconomy by promoting the use of waste products as a valuable resource.

## 1. Introduction

Bioactive compounds are present in small amounts in foods and other natural matrices [1] and provide an extra-nutritional contribution to diet due to their beneficial effects on health. Scientific research has recently been focused on the development of new and more advantageous methods for their extraction from food waste [2,3,4,5,6,7,8,9,10], a potential source of useful molecules, in the attempt to provide the food industry with an efficient, cheap, and environmentally friendly solution for waste disposal.

Apple (*Malus domestica* (Suckow) Borkh) is a type of fruit available all year round and is widespread throughout the world for economic and cultural reasons [11,12,13]. According to a “Food and Agriculture Organization” report, the 2020 world apple production was estimated at ~86 million tons. Approximately 18% of global production was used to produce transformed foods, generating large amounts of by-products whose disposal represents an environmental problem and which add additional costs to food producers [13,14]. However, due to the high content of compounds useful for human health, apple waste constitutes a potential resource [14,15,16,17,18,19,20,21]. For instance, apple peel [21,22] contains fibers [19,21], minerals [18], flavonoids [16,19], phenolic acids [16,19], and vitamins, such as C, B1, B2, B3, and A [23,24], possessing great potential in functional food development and exerting a positive influence on the prevention of various diseases. The distribution of bioactive compounds varies between the peel, pulp, and seed [2,16,25], even if the peel is characterized by higher concentrations, and notable differences can also be observed in fruits of the same cultivar.

Polyphenols, likely the most important bioactive compounds in apple peels, are naturally present as acetyl-, malonyl-, and β-glucosides, conjugated precursor forms characterized by greater hydrophilicity and lower biological activity compared to the molecules that represent their core. The free polyphenolic forms therefore represent their least abundant hydrophobic active fraction [26].

Traditionally, extractions on apple peel were based on conventional techniques, which were neither applicable to large-scale samples nor environmentally friendly, as they used large amounts of organic solvents on fresh or dried peels [20], and nowadays green extraction technologies are preferred to process apple peel into products of added value [2,3,14]. Among them, supercritical fluid extraction (SFE) constitutes a simple, cost-effective, eco-friendly approach, which can even be applied to systems on different scales. In fact, it can be used in laboratories on small amounts of samples for analytical or preparative purposes, or to treat quantities of raw material in the order of kilograms (pilot systems) up to tons (industrial sector) [3,20]. The use of small amounts of organic solvent, such as methanol or ethanol, with supercritical CO_2_ was successfully used for the extraction of polar compounds, such as polyphenols and water-soluble vitamins, allowing the simultaneous recovery of compounds of different polarities [27].

In this study, CO_2_ was used as a supercritical fluid with ethanol as a modifier to extract bioactive compounds from apple peel. The waste apple peels were obtained from three of the most widespread cultivars in Italy, two from organic farming (Royal Gala and Stark) and one from non-organic farming (Gala). Each SC-CO_2_ extract was characterized in terms of total phenolic content, flavonoid, anthocyanin, vitamin C, and antioxidant activity, while chromatographic analyses were also performed to highlight the differences between the extracts.

Since several mitochondrial diseases and mitochondrial dysfunction associated with ageing and some cancers [28] may depend on NADH–ubiquinone oxidoreductase (Complex I) impairment [29,30], it is crucial to find small molecules that are able to stimulate Complex I activity and/or mitochondrial function. Thus, each extract was subjected for the first time to in vitro tests on mitochondria from human embryonic kidney cell lines (HEK293) [31] to evaluate their abilities in stimulating the oxidation of NADH and mitochondrial function for possible future pharmacological applications.

## 2. Materials and Methods

### 2.1. Apple Collection and Apple Peel Preparation

Apples from Stark, Gala, and Royal Gala cultivars were obtained from apple orchards in Italy and acquired by local suppliers in the period August–September 2021. Royal Gala and Stark cultivars were both from Italian organic farms and purchased from local suppliers in the same period.

The fruits were washed with tap water, thin peels were obtained from each cultivar and cut into small pieces, put on Petri plates, weighed, and immediately covered with paper to avoid light exposure. Samples were frozen at −20 °C for 24 h and finally freeze-dried for 48 h in a ScanVac cool safe lyophilizer supplied by Chemie (BA, Italy), operating at a temperature of −50 °C and a pressure of 0.5 torr. Freeze-dried peel samples were stored in a dryer at room temperature in the dark until use.

### 2.2. Supercritical Fluid Extraction

SC-CO_2_ extraction was carried out according to Aresta et al. [5], with modifications. The SC-CO_2_ system was a Spe-ed SFE from Applied Separations (Allentown, PA, USA) connected in-line with a 40P pump from Knauer Wissenschaftliche Geräte GmbH (Berlin, Germany) capable of introducing the modifier into the SC-CO_2_ line via a pressurization branch and a T-valve. One gram of each freeze-dried peel sample was mixed with a small quantity of a dispersing material, Ottawa sand (O_2_Si, 50–70 mesh particle size, Applaied Separation), to facilitate the contact between the supercritical fluid and the sample particles. The mixture was placed into a 10 mL extraction cell, which was sealed under pressure before being introduced into the oven at 60 °C.

Carbon dioxide (supercritical fluid grade, SCF) from Rivoira (Milan, Italy) was used as extraction solvent with 20% ethanol (liquid chromatographic grade, LC; Sigma-Aldrich, Milan, Italy) as co-solvent. Before each extraction, manual purging of the co-solvent pump was necessary to ensure the correct flow at the same stage and to prevent the pump from undergoing a pressure stroke during extraction. Then, a static period of 3 min was applied before starting the extraction by activating the co-solvent pump. Subsequently, the dynamic extraction process was carried out at 60 °C and 250 bar for 15 min, with carbon monoxide and ethanol (0.4 mL/min) at a total flow rate of 2 mL/min. The extracts were collected in amber vials using the heated micrometric valve, set at 70 °C. At the end of each extraction cycle, an ethanol cleaning cycle was carried out using a stainless-steel by-pass instead of the jar, for 3 min, to remove all residues.

The extracts were then filtered through a syringe filter device with a 25 mm diameter and 0.45 μm PTFE (polytetrafluoroethylene) membrane (Sigma-Aldrich) before analysis.

### 2.3. Total Antioxidant Capacity (TAC) Assay

The assay was performed according to slightly modified previous literature protocols [32]. An aliquot (10 μL) of SC-CO_2_ extract (S) was transferred into a test tube containing 0.99 mL of 0.1 mM 2,2-diphenyl-1-picrylhydrazyl (DPPH; Sigma Aldrich) or ethanol (EtOH). The solutions were kept in the dark for 1 h at room temperature. The absorbances were read at 517 nm against EtOH using a UV–Visible spectrophotometer (UV-1650 PC; Shimadzu, Kyoto, Japan). The radical scavenging activity (*RSA*) was expressed as the percentage of the DPPH consumed, which was calculated using the following formula:

$$\%RSA=100-\left[\left(AS-AEtOH\right)÷ADPPH\right]\times 100$$

where *AS* is the absorbance of the extract with DPPH, *AEtOH* is the absorbance of the extract without DPPH, and *ADPPH* is the absorbance of the DPPH solution. The calibration curve was prepared with standard solutions of (±)-6-Hydroxy-2,5,7,8-tetramethylchromane-2-carboxylic acid (Trolox; Sigma Aldrich) in the range 1–100 mM. The TAC was expressed as the Trolox equivalent antioxidant capacity (TEAC) (mmole of Trolox/100 g of freeze-dried material, f.d.m.) [33].

### 2.4. Quantification of Antioxidant Compounds

#### 2.4.1. Total Phenol (TP) Determination

The TP content was evaluated by using 4-benzoylamino-2,5-dimethoxybenzenediazonium chloride hemi [zinc chloride] salt, namely Fast Blue BB diazonium salt (FB BB D; Sigma Aldrich), according to Medina et al.’s protocol [33]. Briefly, 0.1 mL of 0.1% (*w*/*v*) FB BB D reagent in 0.1% ethanol was added at 1 mL of SC-CO_2_ extract previously diluted 1:10 in water. Samples were accurately mixed and 0.1 mL of 20% (*w*/*v*) Na_2_CO_3_ in 1 M NaOH was added to each test tube. The absorbances of samples were measured at 420 nm at 0 and 30 min and the difference between two measurements was used for the estimation. Gallic acid (GA; Sigma Aldrich) was used as the standard compound (calibration curve in the range 0.02–20 μg/mL) for the quantification of TPs, expressed as mg of GA/100 g of f.d.m. Each measurement was performed in triplicate.

#### 2.4.2. Total Flavonoid (TF) Determination

The content of TFs was carried out following the method proposed by Zhishen et al. [34], with a few modifications. Briefly, 50 μL of SC-CO_2_ extract was dried in a test tube and 0.5 mL of distilled water (suitable for HPLC; Sigma-Aldrich) was added to the residue. After accurate mixing, 37.5 mL of 5% (*w*/*v*) NaNO₂ (Sigma-Aldrich) was added to each test tube, which was then kept for 5 min at room temperature. Subsequently, 37.5 mL of 10% (*w*/*v*) AlCl_3_ (Sigma*-*Aldrich) was added. After 6 min, 250 mL of 1 M NaOH was added, mixed, and then each tube received another 300 mL of distilled water. The absorbance at 510 nm of the mixture was finally measured against a blank sample obtained in the same mode. GA was used as the standard compound for the quantification of TFs, expressed as mg of GA/100 g of f.d.m. Each measurement was performed in triplicate.

#### 2.4.3. Total Anthocyanin (TA) Determination

The monomeric anthocyanin content of the SC-CO_2_ extract from apple peel was measured using a spectrophotometric pH differential protocol [35]. An aliquot (0.1 mL) of apple peel extract was mixed thoroughly with 0.9 mL of 0.025 M KCl pH 1 buffer. The absorbance of the mixture was measured at 515 and 700 nm against a distilled water blank. Another aliquot was combined similarly with 25 mM sodium acetate buffer pH 4.5, and the absorbance of the solution was measured at the same wavelengths. Finally, the differences between (A_515nm_ − A_700nm_) pH 1 and (A_515nm_ − A_700nm_) pH 4.5 were used for the determination of total anthocyanins. Cyanidin-3-glucoside chloride (CG, Reference Substance; Sigma-Aldrich) was used as the standard compound for the quantification of anthocyanin content in each apple peel extract. Total anthocyanins were expressed as mg of CG/100 g of f.d.m. Each measurement was performed in triplicate.

#### 2.4.4. Ascorbic Acid (AA) Determination

The quantification of AA was performed according to Kschonsek et al. [22]. Briefly*,* an aliquot (0.2 mL) of SC-CO_2_ extracts, previously diluted 1:10 with ultra-pure water, was mixed with 0.3 mL of trichloroacetic acid (0.31 M; Sigma Aldrich) and centrifuged (5 min, 17,000× *g*). Then, 0.3 mL of supernatant was mixed with 0.1 mL of DNP reagent [one volume of thiourea solution (0.83 M in distilled water), one volume of copper sulfate solution (24 mM in distilled water), and 20 volumes of 2,4-dinitrophenylhydrazine solution (0.11 M in 4.5 M sulfuric acid). All reagents were provided by Sigma Aldrich]. The mixture was heated in a thermomixer for 1 h at 60 °C. The samples were cooled in an ice bath for 5 min. Then, 0.4 mL of sulfuric acid (9 M; Sigma Aldrich) was added and mixed. The samples were kept in the dark for 20 min. Finally, each sample was transferred into a semi-micro cuvette and measured at 520 nm. Each measurement was performed in triplicate. In the same way, a calibration line was obtained with six standard solutions of AA (Reference Standard; Sigma Aldrich) covering the concentration range of 0.06 to 6 mg/mL.

### 2.5. HPLC/DAD Analysis

Chromatographic analyses were performed using an HPLC system (LC20ADXR; Shimadzu, Milan, Italy) equipped with a binary pump (LC20ADXR; Shimadzu), an auto sampler (SIL20ACXR; Shimadzu), and a UV-diode array detector (PDA-1; Shimadzu) with a flow cell (10 mm, 1/1600, steel, 2.4 mL, LWL). The chromatographic column was an Accucore XLC18 (150 × 4.6 mm, 4 μm; Thermo Scientific, Milan, Italy) equipped with an Accucore XLC18 precolumn (10 × 4 mm, 4 μm; Thermo Scientific).

The mobile phase used was (A) 0.1% formic acid in water, (B) acetonitrile (Sigma Aldrich) with a flow rate of 1.2 mL/min. The gradient elution program was as follows: B (8%) (2 min), 8% B to 15% B (3 min), 15% B to 25% B (5 min), 25% B to 35% (5 min), 35% B to 50% B (5 min), B (50%) (15 min), and re-equilibration of the column (5 min). The total chromatographic run time was 45 min. Injection volume was 20 μL.

Spectra were acquired in the range of 220–500 nm, while meticulous composite chromatograms were obtained using the Max Plot software 5.17 of the PDA-1 detector, which automatically selects and stores the maximum absorbances for each peak, thus providing the maximum signal for all compounds detected. The phenolic compounds were identified by comparing their retention times and UV–visible spectrum with those obtained from standard compounds (10 mg/mL; all Sigma-Aldrich), when available. Otherwise, compounds were tentatively identified by comparing the obtained information with the available data reported in the literature.

An enzymatic deconjugation was also performed as follows: 0.1 mL of enzymatic solution for glycoside hydrolysis [obtained by dissolving 10 mg of β-Glucuronidase from bovine liver (type B-1, ≥1,000,000 units/g solid; Sigma Aldrich) in 13 mL of 0.1 M acetate buffer, pH 5.0] was added to 0.05 mL of SC-CO_2_ extract and the resulting mixture was immediately incubated overnight (i.e., approximately 17 h) at 37 °C. Before being subjected to chromatographic analysis, the mixture was filtered through a syringe filter device with a 13 mm diameter (0.45 μm PTFE; Sigma-Aldrich). The experiments were performed in triplicate.

### 2.6. Evaluating Biological Activity

#### 2.6.1. Cell Cultures

HEK293 cells were obtained from the American Type Culture Collection (https://www.atcc.org/products/crl-1573) accessed on 12 September 2022. For the proposed analyses, HEK293 cells were maintained in Dulbecco’s Modified Eagle’s Medium (DMEM), which has high glucose with sodium pyruvate and stable glutamine (Euroclone, Pero (MI), Italy), and supplemented with 10% (*v*/*v*) fetal bovine serum (FBS; Euroclone ECS0180L, Pero, Milan, Italy) and 1% (*v*/*v*) penicillin-streptomycin (Euroclone, *ECM0010*, Pero, Milan, Italy). The cells were cultured in a humidified atmosphere with 5% CO_2_ and 37 °C.

#### 2.6.2. Mitochondrial Isolation

Cells were grown in T75 flasks. At confluence, cells were detached by trypsin and centrifuged at 125× *g* for 5 min, the supernatant was discarded, and the pelleted cells were resuspended in Ringer NaCl buffer (135 mM NaCl, 20 mM HEPES, 0.8 mM MgSO_4_, 3 mM KCl, 1.8 mM CaCl2, 11 mM D-glucose, pH = 7.5) according to Palacino et al. [36]. Afterwards, cells were centrifuged at 125× *g* for 5 min and the obtained pellet was suspended in 2 mL of A buffer (sucrose 320 mM, Tris-HCl 5 mM, EGTA 2 mM, pH = 7.4) and homogenized with a glass–Teflon grinder kept in ice. Thus, the homogenate underwent a centrifugation step at 4 °C for 6 min at 2000× *g* to remove nuclei and tissue particles, while the supernatant was collected for further centrifugation at 4 °C for 15 min at 12,000× *g* to pellet mitochondria. The entire isolation procedure of subcellular fractions was carried out according to Palacino et al. [36]. The mitochondria amount was determined according to Bradford methods [37].

#### 2.6.3. Complex I Activity Measurements

Rotenone-sensitive NADH (β-Nicotinamide adenine dinucleotide) ubiquinone oxidoreductase (Complex I) activity was measured spectrophotometrically following the decrease in NADH absorbance at 340 nm as described by Spinazzi et al. [38], with some modifications. We used a reaction mix containing a 25 μg sample of mitochondria, TNS buffer 3% (*w*/*v*), 50 mM potassium phosphate buffer pH 7.5, 600 mM KCN (Sigma Aldrich), and 90 mM NADH (≥97% pure, grade HPLC; Sigma Aldrich). The mitochondria were incubated for 2 min with apple peel extracts diluted 1:100, 1:1000, or 1:10,000 in H_2_O or with only ethanol used as a control (at the same dilutions in water) before starting the reaction. The reaction was started by adding 30 mM Decilubiquinone (≥97% pure, grade HPLC; Sigma Aldrich). NADH (β-Nicotinamide adenine dinucleotide) ubiquinone oxidoreductase (Complex I)-specific activity was checked by adding an excess of Rotenone 100 μM. In parallel, as a further control test, we prepared a cuvette containing the same quantity of extract (ethanol) dilutions and the mitochondrial sample in the presence of 100 μM Rotenone (≥95% pure; Sigma Aldrich), which is a Complex I selective inhibitor.

#### 2.6.4. Statistical Analysis

All analyses related to the quantification of antioxidant compounds were performed in triplicate and the results expressed as mean values ± standard deviation. MedCalc statistical software ver 22.021 for Windows was used to calculate the difference between the averages observed in two independent samples. A significance value (*p* value) < 0.05 was used to assess the difference. For NADH–ubiquinone oxidoreductase activity in HEK cell mitochondria, a nonparametric Wilcoxon test was used to calculate the difference (*p* < 0.05) between mitochondria incubated with diluted SC-CO_2_ extract diluted from Stark, Gala, and Royal Gala wastes and nontreated mitochondria in the corresponding ethanol obtained from at least four independent experiments.

## 3. Results and Discussion

### 3.1. Supercritical Fluid Extraction

The parameters applied during supercritical fluid extraction (SFE) were found in the literature [1,2,3,4,36]. Ethanol with a percentage of 20% was selected as the extraction co-solvent to help CO_2,_ which has a low polarity, to favor the extraction of compounds with different polarities from apple peel [5,27].

Additionally, a mild temperature (60 °C) was selected to avoid thermal degradation of the compounds and preserve the antioxidant properties of the extracts [4,36]. Given these observations, the samples were prepared according to the protocol optimized by Aresta et al. for the determination of polyphenols and vitamins in winemaking by-products by extraction with SC-CO_2_ [5].

### 3.2. Quantification of Antioxidant Compounds

The specific spectrophotometric assays for the determination of the total antioxidant capacity and the quantification of the antioxidant compounds present in the extracts of Stark, Gala, and Royal Gala apple peels obtained by SFE (Section 2.2) were selected from the literature [33] considering specific features, such as sensitivity, specificity, and ease of execution. TPs were detected using the FB BB D reagent, which, unlike the more common Folin–Ciocalteu reagent, is not sensitive to reducing sugars which can be naturally present in extracts and determine overestimates with Folin–Ciocalteu [33].

Table 1 shows the average values of the complete set of determinations (TAC, TPs, TFs, TAs, and AA) performed on each apple peel SC-CO_2_ extract. The data obtained were in good agreement with the existing literature for all the analytes except for ascorbic acid, which was found in all extracts at concentration levels lower than expected, since Kschonsek et al. [22] reported that peel levels for TPs and AA range from 99.6 ± 5.4 to 495.3 ± 44.0 mg/100 g and 99.2 ± 10.0 to 300.9 ± 10.8 mg/100 g, respectively.

T-tests showed some statistically significant differences between the extracts of the three cultivars in the five assays (*p* < 0.05). Stark extracts were higher in TPs, TAs, and AA than the other two. Instead, they showed no significant differences in comparison with Royal Gala for TAC. Gala and Royal Gala were similar in TP and TA. On the other hand, vitamin C contents were very different between the three extracts. In particular, the Gala apples were characterized by lower levels and lower antioxidant activity. Then, phytochemicals can contribute in different ways to the antioxidant activity of the extracts and the poor correlation between the estimated amounts of the analytes and the antioxidant power of the samples.

### 3.3. HPLC/DAD Analysis

**Figure 1:**
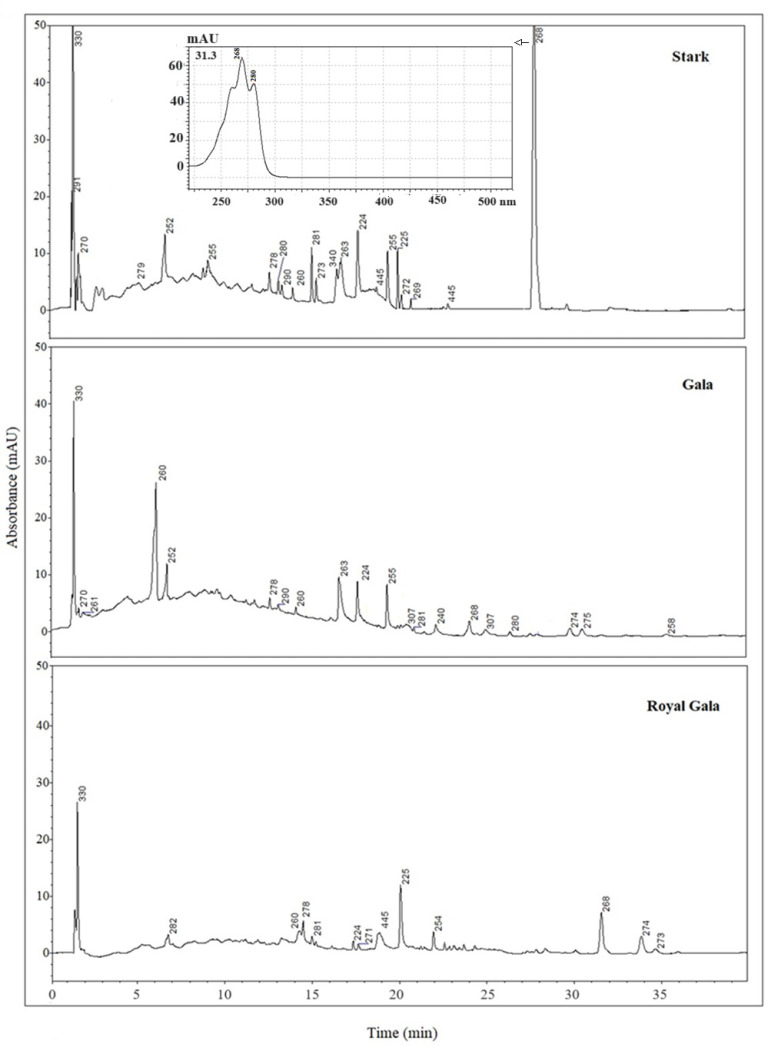
HPLC/DAD chromatograms (max plots) of SC-CO_2_ extract from the apple peel of Stark, Gala, and Royal Gala cultivars. The numbers refer to the λ_max_ detected for each peak. Chromatographic and acquisition conditions are described in Section 2.5.

Chromatographic analyses confirmed the existence of differences between samples deriving from apple waste of different origins. Figure 1 shows chromatograms relevant to the three SC-CO_2_ extracts (Stark, Gala, and Royal Gala). The chromatograms appear rather poor, although apple peel is a rich source of polyphenols.

However, some polyphenols were identified by comparing both the chromatographic retention times and the UV–Visible spectra taken at the apex of each peak with those obtained from standard compounds analyzed under the same conditions.

Table 2 lists the standards that were subjected to chromatographic analysis, their retention times (RT, min), the λ_max_ recorded by the detector, and the presence or absence in the considered cultivar.

For analyte recognition, the purity of each peak was determined. The spectra acquired at the apex and in the ascending and descending tracts of each peak during elution were superimposed with the spectrum of the standard acquired in the same peak, always obtaining results better than 99%.

Rutin is a glycoside of the flavonol quercetin, in which the aglycone is bound to the disaccharide α-L-rhamnopyranosyl-(1→6)-β-D-glucopyranose. It is found in a wide variety of plants and apple peels and is believed to be a rich source of flavonoids [8]. However, it was unexpectedly detected only in Stark extracts.

**Figure 2:**
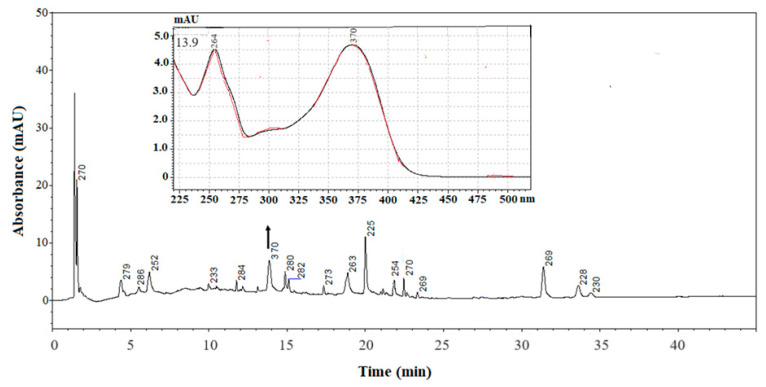
LC-UV-DAD chromatograms (max plots) of enzymatically hydrolyzed SC-CO_2_ extract from the apple peel of the Stark cultivar. The numbers refer to the λ_max_ detected for each peak. The figure shows the spectrum of the compound at 13.9 min, coincident with quercetin standard (red line). Chromatographic and acquisition conditions are described in Section 2.5.

Then, all the SC-CO_2_ extracts were subjected to enzymatic hydrolysis to obtain the respective aglycones. The glycosylated deconjugates were then subjected to LC analysis in order to check their presence. Figure 2 shows, for instance, an LC chromatogram of a deconjugate Stark sample. Both the retention time and the UV–Visible spectrum of the peak eluting at 13.9 min were found to be clearly comparable to those of the standard of quercetin analyzed using the same chromatographic conditions. On the contrary, analysis of the deconjugates related to the other two cultivars confirmed the absence of the flavonol.

To evaluate the quercetin levels in the sample, a calibration curve was generated with five standard solutions that was linear in the range 0.06–20 μg/mL, with a determination coefficient (R^2^) of 0.9998. The detection and quantification limits obtained by interpolation of the calibration curve were 0.02 and 0.06 μg/mL, respectively, according to European Commission recommendations [39].

The method had good repeatability, with RSD values of 5.9 and 9.7% for within days (n = 3) and between days (n = 15), respectively. In relation to the concentration of quercetin in deconjugated samples, it was undetectable at 50 μg/mL.

The careful inspection of retention times and spectra and the provisional identification, also compared with the obtained information from the literature data, showed the probable presence of catechins (λ_max_ 280 nm), caffeic acid (238 nm), cyanidins (278 nm), chlorogenic acid (324 nm), isoflavones (260 nm), retinoid (340 nm), and carotenoids (445 nm) [40,41].

In the chromatograms relative to the SC-CO_2_ extracts of Stark samples, the presence of a very abundant peak is evident at the retention time of 31.3 min, whose UV–Visible spectrum is reported in Figure 1. Interestingly, this spectrum is shared with other peaks of the same chromatogram, namely the one with RTs of 18.9, 22.5, and 23.4 min. Comparing the chromatographic data relevant to this extract with those of the other cultivar analyzed, only one peak in the chromatogram relevant to the Royal Gala extract showed a peak eluting at 18.9 min, also characterized by the same UV–Visible spectrum. It is worth noting that the Royal Gala and Stark cultivars were both from organic farms.

### 3.4. Evaluating Biological Activity

To examine the effect of each apple peel extract on mitochondria, the NADH oxidoreductase activity was measured on mitochondria isolated from HEK293 cells in the presence of different dilutions of the investigated extracts.

**Figure 3:**
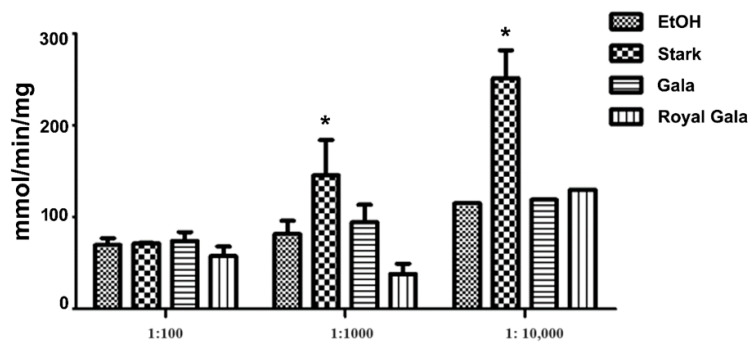
NADH–ubiquinone oxidoreductase activity in HEK cell mitochondria incubated with SC-CO_2_ extract diluted from Stark, Gala, and Royal Gala waste. Values represent mean rates (nmol/min/mg) ± SEM obtained from at least four independent experiments. * *p* < 0.05, nonparametric Wilcoxon test between mitochondria administered with 1:100, or 1:1000, or 1:10,000 and nontreated mitochondria in the corresponding ethanol (EtOH). Dilution in water (1:100, 1:1000, or 1:10,000).

Firstly, it was noticed that the Complex I activity measured from mitochondria extracted from the cultured selected cells after the addition of the three apple peel extracts at 1:100 dilution was unchanged with respect to the ethanol control. Instead, we observed an increase in Complex I activity in the presence of the Stark apple peel extract at 1:1000 and above all at 1:10,000 dilution, with respect to the ethanol control at the same dilution, whereas the Complex I activity in the presence of Gala and Royal Gala apple peel extracts was unchanged (Figure 3).

As is evident, ethanol does not affect mitochondrial function, as observed by Tempio et al. [42], while only SC-CO_2_ Stark extracts were able to induce a significant stimulation of Complex I activity at higher dilutions, probably due to their higher concentration of bioactive molecules (almost double the concentration of AA, TP, and TA) compared to the other cultivars studied. Furthermore, the increased Complex I activity in the presence of diluted Stark extracts could be ascribed either to the minimum concentration of bioactive molecules able to produce a positive effect without causing any toxicity, or to a combination of AA, TPs, and TAs able to exert a protective effect on mitochondria at specific concentration levels.

Moreover, Stark extracts were well tolerated by mitochondria, as well as comparable to the other investigated extracts, even if an excess of antioxidant molecules, which may counteract ROS formation into mitochondria, can alter the endogenous ROS signaling between the cytosol and mitochondria, and can even result in toxicity [43,44]. Indeed, apple peels are rich in vitamins (i.e., vitamin E and vitamin K [45,46]) that can be structurally related to ubiquinone and can target either mitochondrial proteins of the inner membrane or other FAD/NADH/ubiquinone-dependent dehydrogenases widely expressed in other cell compartments [43].

More generally, the hypothesis of a protective role of the tested extracts on mitochondrial function is consistent with Carrasco-Pozo [47], who proposed a protective role of dietary polyphenols on mitochondria. It was also shown that quercetin, resveratrol, and rutin protected Caco-2 cells against indomethacin-induced mitochondrial dysfunction due to the ability of the molecules to enter cells and accumulate in mitochondria. In addition, a structural similarity between quercetin and ubiquinone has been proposed by some authors as a possible explanation for the protective effect [45]. Furthermore, Skemiene et al. [48] proposed that anthocyanins can behave as electron acceptors in Complex I, resulting in increased ATP production in pathological conditions, such as after ischemia.

Based on the experimental results obtained, which are coherent with analogous findings described in the literature, Stark apple peel extracts appear to be a potentially advantageous dietary supplement for human health. Thus, they will be the subject of further studies aimed at determining the individual polyphenol concentrations. The role of each compound in terms of antioxidant capacity and in terms of protective activity on mitochondria in the presence of pro-apoptotic molecules will be explored by performing mitochondrial respiration assays on mitochondria extracted from different cell lines.

## 4. Conclusions

In this work, it has been shown that bioactive molecules can be extracted simultaneously from apple peels by means of SFE. In particular, the waste apple peels obtained from an Italian cultivar from an organic farm (Stark) seem the most promising both for the higher content of total polyphenols, anthocyanins, and vitamin C, but also for the greater presence of lipophilic substances, the nature of which is being investigated. The ratio between the constituents, however, is the most balanced among the three investigated to preserve or stimulate mitochondrial function without inducing any toxicity.

## Figures and Tables

**Table 1:** TAC, TPs, TFs, TAs, and AA apple peel SC-CO_2_ extract contents. For every cultivar, three replicates were performed (n = 3).

Cultivar	TAC mmole Trolox/100 g (n = 3)	TPs mg GA/100 g (n = 3)	TFs mg GA/100 g (n = 3)	TAs mg CG/100 g (n = 3)	AA mg/100 g (n = 3)
**Stark**	5.74 ± 0.07	379 ± 68	120 ± 2	7.0 ± 0.3	43.75 ± 6.05
**Gala**	2.20 ± 0.04	152 ± 25	90 ± 1	5.0 ± 0.3	9.02 ± 1.65
**Royal Gala**	5.95 ± 0.89	136 ± 14	115 ± 2	4.8 ± 0.1	25.59 ± 1.96

**Table 2:** Retention times of standard compounds, λ_max_ detected, and presence in SC-CO_2_ extract.

RT (min)	Compound	λ_max_ (nm)	Presence in SC-CO_2_ Extract
2.0 ± 0.1	Gallic acid	270	Stark and Gala
5.0 ± 0.1	(+)-Catechin	279	Stark
5.6 ± 0.1	Vanillic acid	260	Gala
9.1 ± 0.1	Rutin	255	Stark
12.4 ± 0.1	trans-Resveratrol	307	
13.8 ± 0.1	Quercetin	370	
14.3 ± 0.2	Cinnamic acid	278	Royal Gala

## Data Availability

The data presented in this study are available on request from the corresponding author.
